# Assessing the Impact of the Twin Track Socio-Economic Intervention on Reducing Leprosy-Related Stigma in Cirebon District, Indonesia

**DOI:** 10.3390/ijerph16030349

**Published:** 2019-01-26

**Authors:** Dadun Dadun, Ruth M. H. Peters, Wim H. van Brakel, Joske G. F. Bunders, Irwanto Irwanto, Barbara J. Regeer

**Affiliations:** 1 Center for Health Research, Faculty of Public Health, Universitas Indonesia, Building G, Room 211, Depok 16424, Indonesia; 2 Centre for Disability Studies, Selo Sumarjan Research Centre (SSRC), Faculty of Social and Political Sciences, Universitas Indonesia, Depok 16424, Indonesia; irwanto_i@yahoo.com; 3 Athena Institute, Faculty of Science, Vrije Universiteit Amsterdam, De Boelelaan 1085, 1081 HV Amsterdam, The Netherlands; r.m.h.peters@vu.nl (R.M.H.P.); j.g.f.bunders-aelen@vu.nl (J.G.F.B.); b.j.regeer@vu.nl (B.J.R.); 4 Disability Studies, VUmc, De Boelelaan 1117, 1081 HV Amsterdam, The Netherlands; W.v.Brakel@leprastichting.nl; 5 NLR, Technical Department, Wibautstraat 137k, 1097 DN Amsterdam, The Netherlands

**Keywords:** leprosy, stigma, disability, socio economic, twin track approach, Indonesia

## Abstract

The consequences of leprosy go beyond the physical, social and psychological, as leprosy can drive persons affected and their families into poverty, stigmatization and disability. This paper describes the impact of a socio-economic development (SED) intervention that uses a twin-track approach (two micro-credit models) to reduce leprosy-related stigma in Cirebon District, Indonesia. A randomized-controlled mixed-methods study design was used to test the effectiveness of the SED intervention. Three scales were used to measure stigma and participation restrictions among 30 SED clients and 57 controls, 20 in-depth interviews with SED clients and seven Focus Group Discussions (FGDs) with key persons were held and 65 profiles of the clients were written up and analysed. The qualitative data shows the socio-economic status of 44 out of 65 SED clients (67%) improved. The median family income increased by 25%, more clients reported higher self-esteem, better interaction with neighbours and less stigma than before, although disclosure concerns remained an issue. The scales indicate a positive effect of the intervention on reducing stigma (e.g., Stigma Assessment and Reduction of Impact (SARI) stigma scale mean difference total score of pre and post assessment for SED clients versus the control group was 8.5 versus 5.6). A twin track socio-economic intervention, if embedded and integrated, can increase participation, and be constructive in reducing leprosy-related stigma.

## 1. Introduction

Stigma plays a significant role in a variety of conditions and diseases, including HIV/AIDS, mental illnesses, tuberculosis, Ebola and leprosy [1,2,3,4,5]. Health-related stigma is an important problem because it has various adverse and interconnected effects on the person affected—physical, social and psychological. In the case of leprosy, the physical impacts such as a deformity can cause or increase stigma towards people affected, reducing their self-esteem, which results in negative feelings and emotions [6,7,8]. The impact of stigma goes well beyond the individuals directly affected by the condition or disease. Health professionals, families, social networks, the wider community and others associated with an affected person are often also stigmatized, a process called courtesy stigma [9]. Leprosy can drive persons affected and their family into poverty or deepen their existing poverty.

The relationship between leprosy, stigma and poverty has been explored by various scholars [7,10,11,12]. Leprosy can lead to stigmatization, which underpins two negative dynamics. First, stigmatization is usually followed by social rejection, exclusion and discrimination, and sometimes even by family members and loved ones [3,13,14,15,16]. Second, people affected by leprosy sometimes become isolated because of a fear of infecting others or because of internalized feelings of being less worthy [3,14,15,16]. As a result, people affected by leprosy might lose their job or their customers, or may decide to resign or close their business. This affects the financial situation of their household [17]. In a way there is a double burden: Poverty and leprosy both produce stigma. Even worse, if the people affected by leprosy delay seeking care, for example due to poverty and stigma, they might develop an impairment which might lead to disability and even more stigma and poverty [18]. 

Previous studies show that people affected by leprosy see benefits in a socio-economic intervention [19]. In one of our previous studies a participant affected by leprosy made a direct link between running a business and less mockery (a manifestation of stigma). She said, ‘*… to avoid mockery, we have to run a business*’ [19]. A key person in the same study suggested that financial aid of some sort ‘*could give them (people affected by leprosy) the passion to live their life again*’. Similarly, Velema et al. mention that clients of a socio-economic intervention can have an enormous boost in self-esteem [20]. They also note that activities such as selling products at the market changes dynamics among people in their community with the result that they might perceive people affected by leprosy more positively [20]. Recent studies show similar effects. Abera and Shanko [21] found in their study on leprosy in Ethiopia that 86% of the participants who received a small loan reported a considerable improvement in attitudes of community members and family members. Likewise, Ebenso et al. concluded from their study in Nigeria that socio-economic rehabilitation can lead to more social interaction and change community attitudes [11]. 

Socio-economic intervention succed to improve well being and economic life of the people affected by leprosy, but at the same time faced important challenges for creating a sustainable socio-economic intervention for people affected by leprosy [14,22,23,24]. Challenges include certain characteristics of people affected (e.g., low self-esteem, shame, internalized stigma), people’s ignorance about leprosy, and negative views in society that may affect the business. As a result, there are serious doubts whether a local financial or social organization would accept leprosy clients as a target group for microcredit. The rationale is that setting up a business with microcredit is difficult enough without the additional barriers of being affected by leprosy; giving loans to such people is considered too much of a risk. (Indeed, often socio-economic programmes for people affected by leprosy revolve around providing grants instead of loans, e.g., [10,25,26], making socio-economic rehabilitation expensive and unsustainable.) At the same time, in order for a socio-economic development (SED) intervention to be sustainable in the longer term, working with established local actors/parties and using an integrated approach are seen as a key prerequisite [27,28]. This poses a challenge to the current project. Sustainability should be a ‘built-in’ criterion of the intervention design. Some concrete suggestions on how to deal with these challenges are also provided in the literature. Even more than in standard microcredit programmes, those aimed at people affected by leprosy Abera and Shanko [21] highlight the importance of investing in a good relationship between them and the credit provider (e.g., regular follow-up visits, advice). In addition, Ebenso et al. [11] describe the importance of involving key persons in the community, connecting to local government bodies, and including people with general disabilities. It is thus not straigthforward to develop a sustainable socio-economic intervention that can deal with these challenges and that contributes to earning an income and reducing reduction. A combination of an exploratory study, baseline study, implementation phase and final survey was carried out to understand the development and effect of the intervention. 

The aim of this study is to assess how effectively a twin track of SED intervention can improve socio-economic status, reduce leprosy-related stigma, and facilitate social transformation in people affected by leprosy in Cirebon District, Indonesia. A secondary aim is to explain the factors that contributed to the observed changes, giving insight into how the intervention accommodates the challenges of developing a SED intervention that is sustainable after the end of the project. 

### Theoretical Framework: Stigma and Socio-Economic Transformation 

In this study we use the conceptualization of stigma by Weiss [29] to distinguish between different types of stigma so we are able to see where an SED intervention precisely has an effect. In addition, we use the model of Pati and Lyngdoh [24] to show how a microfinance intervention can lead to economic and social transformation and which indicators are important.

To distinguish between different types of stigma, Weiss extended the hidden distress model of Scambler [29], contrasting the stigmatized and stigmatizers with different types of stigma in each. In the stigmatizers, Weiss distinguished between enacted, endorsed and accepted stigma, and found internalized, perceived, and experienced stigma [29]. Perceived stigma refers to a person with a stigmatized condition who behaves or feels a certain way due to anticipated responses. Internalized or self-stigma is the stigma people feel because of negative views about themselves, which could lead to feelings of shame and guilt. Finally, enacted stigma is often called discrimination. 

**Figure 1:**
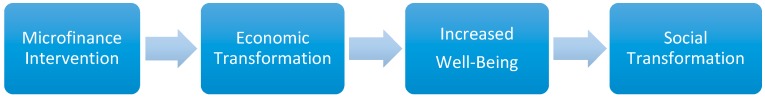
Socio-Economic Transformation Model [24].

A model developed by Pati and Lyngdoh (see Figure 1) shows how a microfinance intervention can lead to economic and social transformation [24]. Economic transformation is described, among other benefits, as having access to an income and savings, economic decision-making and household property. A positive economic transformation can result in increased well-being, which is the start of a ‘social transformation’, which refers to changes in the following indicators: satisfaction (e.g., life vision, planning the future), capacity (e.g., networking, socialization, life skills), decision-making, health, travel and mobility, recognition and acceptance [24]. 

## 2. Materials and Methods 

### 2.1. Study Design and Study Area

This study is part of the Stigma Assessment and Reduction of Impact (SARI) project, which aims to assess the effectiveness of three stigma-reduction interventions. Here we focus on the SED intervention, for which a cluster-randomized controlled intervention study design was selected. A mixed methods approach was used to measure the impact of the intervention on economic and social aspects such as stigma, participation and quality of life. A baseline survey was conducted at the end of 2011 and a final survey in early 2014, allowing us to assess impacts two years after the start of the intervention. The research team comprised senior researchers, PhD students and local research assistants. This study was conducted in Cirebon District, West Java, Indonesia. 

### 2.2. Study Population 

The main study population comprises persons affected by leprosy who participated in the SED intervention, those who participated in other stigma-reduction interventions (counselling and contact, for more information see [30,31,32] and a control group that was not involved in an intervention. Key informants such as family members of SED participants, health professionals and microcredit providers were also part of the study population. 

### 2.3. Research Methods and Sampling 

Assessing a complex concept such as stigma is challenging and the quality of the evaluation depends, among other aspects, on good qualitiative and quantitative methods [33,34,35]. A quantitative and qualitative methods were used to assess the impact of the SED intervention. Each method assesses the socio-economic status and/or stigma and its impact and together we expected them to provide a comprehensive picture. Different sample techniques were used for the different research methods. 

#### 2.3.1. Quantitative Measures 

First, the demographic information of people affected by leprosy was collected. Variables included sex, age, education, marital status and disability grade. To assess socio-economic status, work status and income details were collected. Data on the repayment of the loan that people affected by leprosy received were obtained through the annual reports of the implementing organizations. Socio-economic development interventions are expected both to improve socio-economic status of participants and to increase well-being. In order to study well-being we applied three scales to measure changes in stigma, participation restrictions and quality of life (see Table 1).

The SARI project established a cohort to assess the impact of stigma-reduction interventions and this paper studies a small sample of this cohort (the SED participants). The sample size for the cohort study was based on the requirements for the quantitative comparison of stigma-related scores between the intervention and the control areas. For the calculations, we have used the participation score (P-score), since this measure had the largest standard deviation in the SARI Pilot Project that took place in India and thus would provide the most conservative sample size estimate. The same principles would apply for the other scores also. The mean P-score in Tamil Nadu was ~20 (SD 19) (unpublished data of original validation study). The normal cut-off—the 95th percentile of the P-score among non-affected people—was 10. To reduce the expected mean score (20) to the cut-off for normal (10), a sample size of ~60 subjects was required in each group (SD 20; α-level of 0.05; power 80%). Because of the cluster trial design, we factored in a design effect of 2. I.e., the sample size per group was aimed to be 120. Assuming a loss to follow-up of 20% over 2 years, each comparison group therefore aimed to comprise ~150 subjects. We tested 3 interventions separately and one control area and therefore needed 600 subjects in total. In total 30 sub-districts were randomly allocated a paired intervention (‘SED-Counselling’, ‘SED-Contact’ or ‘Contact-Counselling’) or became control area. Data of people affected by leprosy was provided by the District Health Office (DHO). Only interviews undertaken in Bahasa Indonesia were included. All people affected by leprosy (including those who spoke a language other than Bahasa Indonesia, but excluding those living in a control area) were offered participation in the interventions of the SARI project. During the final survey the scales were repeated. 

#### 2.3.2. Qualitative Measures 

Four qualitative methods were used to assess changes clients experienced in aspects related to economic transformation, well-being and social transformation and the sustainability of the intervention (see Table 1). The participants of in-depth interviews (IDIs) and Focus Group Discussions (FGDs) were purposively selected, aiming for diversity in terms of age and sex. 

### 2.4. Analysis

While analyzing the quantitative and qualitative data we followed the different stages of the model of Pati and Lyngdoh [24]; we start with economic transformation, followed by well-being and finally social transformation. 

Quantitative data was entered using EPI Info versions 3.5.4 (Centers for Disease Control and Prevention., Atlanta, GA, USA) and transferred into Stata version 12.1 (StataCorp LP., College Station, TX, USA) for analysis. Descriptive measures such as means, median, interquartile range (IQR) standard deviation (SD), 95% confidence interval (CI) were used to describe the data. Paired t-tests were conducted to evaluate significance between pre- and post-interventions. A *p*-value of 0.05 was considered significant since some items/domains are more relevant for an SED intervention than others. For instance, we expect a larger change in the internalized stigma domain of the SARI Stigma Scale (SSS) scale compared to the experienced stigma domain. 

For the interviews and FGDs, this study applied content and thematic analysis. First, a contextual text segmentation was conducted to identify similar concepts using open-ended codes. Second, similar concepts were gathered into similar content themes. Finally, triangulation among methods was performed and all the interviews were reviewed to validate the content and to minimize the risk of bias. Observation data from profile participants was used to describe the minimal change of stigma and socio-economic improvement. For the analyses of the profiles, SED participants were grouped by high, moderate and low/no stigma (using indicators such as fear, shame, and interaction with neighbours). The family’s economic condition was based on the ability to fulfil daily needs such as food and paying children’s school fees. The business condition was based on its size and growth.

### 2.5. Overview of the SED Intervention and How It Is Embedded in the Local System

Following our discussion of the challenges associated with SED interventions for people affected by leprosy, we can conclude that simply including these people in microcredit programmes without any adjustments is not appropriate. At the same time, mainstreaming people with a disability into regular services is the dominant discourse in contemporary practice [46,47,48] and contributes both to their inclusion and to sustainability. Given the specific needs of people affected by leprosy, a ‘twin-track’ approach is often promoted [49], and the SED intervention that resulted from experimentation and implementation in the SARI study is characterized by such an approach and a portfolio of activities embedded in different organizational settings.

The so-called ‘twin-track’ approach is a combined approach that is both disability-specific (it addresses special needs) and aimed at mainstreaming disability in general development (it treats disability as a cross-cutting issue). It was developed in the United Kingdom by the Department for International Development (DFID) to achieve greater equality for women [49]. The framework was also perceived to be very relevant for the work related to disability and has been widely adopted by development and disability-related organizations and translated into their activities. It is believed that the twin-track approach can ‘help provide an enabling environment for people with disabilities to achieve greater livelihood security, greater equality, full participation in the life of the community, and more independence and self-determination’ [49]. In the field of leprosy, a ‘twin track approach’—‘in which on the one hand, people affected by all kinds of disabilities receive mainstream services and are involved in mainstream development, while on the other hand special programmes are developed for people with particular types of disability where special needs are identified’—was recommended by Cornielje et al. ([50], p. 31).

The twin track approach in SARI project tried to mainstream people affected with leprosy into existing microfinance businesses, collaborating with Koperasi Mitra Dhuafa (KOMIDA) and the local government. At the same time an alternative microfinance and capacity-building activity was started through a Disability People’s Organization (DPO), Forum Komunikasi Difable Cirebon (FKDC). Three other smaller activities were started; livestock and skills training, both in collaboration with the District Social Welfare Office (DSWO), and individual loans from a family member or friend with individual support through the DPO. Table 2 gives an overview of the five types of socio-economic activities.

The KOMIDA model is based on the familiar Grameen system developed in Bangladesh [51,52]. To become a KOMIDA member, candidates should register and propose how to use the money. Then KOMIDA assesses the financial situation of the participants and what business they would like to run. Microcredit is given in groups of five to ten members following a five-day (10 h) training to build commitment (e.g., building trust) and develop a business plan, followed by weekly meetings to repay the credit and provide support. If one member fails to repay the other group members should cover it. KOMIDA clients are also obliged to join a savings group. KOMIDA charges interest, which helps to pay for managing the microfinance scheme. It also charges 1% of the total credit for life insurance, to ensure the family will not have debts if a participant passes away. Anticipating the challenges of including people affected by leprosy in the programme, the SARI team offered training about leprosy (medical and social aspects) to KOMIDA. People affected by leprosy who lived in areas in Cirebon District in which KOMIDA worked were invited by KOMIDA staff and the research assistants to start or join groups. These were not groups exclusively comprising people affected by leprosy, but there were usually one or two persons affected by leprosy in each. 

FKDC started an alternative model for microfinance and capacity building. FKDC had recently moved from being an informal organization to a formal one. FKDC staff received training from SARI and Community Based Rehabilitation (CBR) consultants on running a microcredit programme and on personal development skills. The difference between the FKDC and the KOMIDA models is that the former is less regulated/strict and that there were monthly rather than weekly repayment meetings. In addition, there is a quarterly gathering of members of the DPO and microcredit participants for coordination and mentoring. The process is simpler than KOMIDA’s since the participants just have to apply for microcredit and propose how to use the money. The repayment is explained in only one day. The FKDC team assesses the (financial) situation of participants, what business they run, and the condition of the neighbourhood. FKDC prioritizes people affected by leprosy and with disabilities; people with leprosy represent about 60%, with a disability 20% and general no more than 20%. FKDC staff know the beneficiaries personally and provide support during the process. FKDC also charges interest to help to pay for managing the scheme. 

Three other smaller socio-economic related activities were started. First, there was an individual support model. The SARI team found that some people affected by leprosy felt insecure about obtaining credit from a formal institution. They were afraid of failing to repay it and being drawn into debt. In view of this, the SARI team encouraged them to have microfinance support from their trusted friends, parents, brother, or other relatives. Together an outreach worker, the participant and the trusted sources would identify the type of business and discuss the possibility of executing the plan. The business started when the trusted sources fund agreed with the proposed business. Second, together with DSWO, which has a programme to support people affected by a chronic disease, started to provide financial or material support (usually in the form of an in-kind grant, for example a goat) to people affected by leprosy. Third, also in partnership with DSWO the SARI team provided skills training for income generation (e.g., sewing, handicrafts, making brooms, electronics). 

Factors for sustainability, such as embedding the intervention in the local context, were in place. In addition, we observed a large interest in, and demand for, the socio-economic intervention from the start. Below we describe the characteristics of the participants. 

### 2.6. Ethics

The study was approved by the ethics committee of Atma Jaya University in Jakarta No.1586/III/LPPM-PM.10.05/2010, and permit from Indonesia Ministry of Health. Informed written consent was obtained from all study participants. The study team guaranteed the confidentiality of the data they provided. 

## 3. Results

### 3.1. Participants of the SED Intervention

About 369 people were involved in the different SED-related activities, including 110 affected by leprosy, 251 community members and eight persons with a disability. In total, 66 persons affected by leprosy accessed 71 units of microcredit (some received more than one loan). Forty people received microcredit from KOMIDA, 20 from FKDC and 11 from a friend or relative. Also, 21 persons affected by leprosy received a goat and 52 participated in different skills training (see Table 3). Most SED participants already had their own business or had done so in the past. From the 325 SED participants, 34% used their credit to work as a food vendor, 24% to open a small shop, 23% to work in farming or selling farm produce, 11% to sell clothes and the other products and 8% for varied activities such as crafts, servicing electric goods or motor cycles, and recycling waste. Most SED participants repaid their loans, 78% within the agreed timeframe, 19% with some delay, and only 3% failed to repay. 

### 3.2. Characteristics of the Study Population

After the baseline and final survey, the SARI project had a cohort with paired data of 237 persons affected by leprosy. Among these, 29 (12%) were SED participants, 57 were controls (24%) and 57 (24%) were from the counselling/contact area (see Table 4 for the demographic characteristics of the respondents), the remaining lived in the ‘SED Contact’ or ‘SED Counselling’ area but did not receive SED. Among the SED participants there were less males compared to the controls and the ‘Counselling Contact’ area (45% compared to 65% and 67%). In total, 74 profiles were written, but only 65 were complete and of use to the analysis.

### 3.3. Economic Transformation: Impact of SED on Socio-Economic Status of the Clients

We found an improvement of about 33% in the median family income following the intervention (from IDR 750 thousand to IDR 1 million). The mean improvement among SED participants was higher (IDR 554,000) than among people in the control area (IDR 363,000) and in the contact/counselling area (IDR 175,000) (see Table 5).

The profiles show a general improvement in the socio-economic condition of the family and the participants’ businesses. Forty-four (67%) have improved family economic conditions since joining the SED activities. They can, for instance, buy more food. The profiles also show that the family’s socio-economic condition in 14 participants (22%) remained stable and in seven (11%) declined. Thirty-nine reported an improvement since receiving microcredit in terms of more sales and more customers; 22 reported stable business conditions or no big changes and four failed to set up or improve the business due to sickness, death, or having a baby.

The analyses of the interviews and FGDs show that SED participants used their loan for starting or strengthening their businesses and so earned more money and reported improved well-being. The data also showed that many SED participants joined saving activities related to the microcredit scheme, improved their assets (e.g., TV set, closet) and property, and had more decision-making power on household issues.

*We can use [the profit] for daily needs and for school… My wife can open a business. We opened a Rujak [traditional fruit and vegetable dish] shop.*

(FGD 2 client SED)

Interviewer: *How much income did you get from that IDR 200,000 capital?*

Interviewee: *Yah, IDR 50,000 [US$ 4]*

Interviewer: *You got IDR 50,000? [US$ 4]*

Interviewee: *If it’s afternoon I earn IDR 25,000 [US $2].*

Interviewer: ... *So if its morning and afternoon you got IDR 75,000 [US $6] per day, right, is it enough for your family daily needs?*

*Interviewee: Thank God, it’s enough.*

Interviewer: *Is it the same between now and before, sir?*

Interviewee: *It’s different.*

Interviewer: *What is the difference, sir?*

Interviewee: *Yes, capital increased, more progress.*

(IDI S-CS18-Man-50)

*I became happier. I have more money. I can go to the market to sell something. I used to only stay at home and have no activity. I have a capital so I can go to the market. So I have a happy feeling.*

(FGD 1 client SED)

*I save at school [microcredit group meeting] ...I save at home too. I save the change from the money spent on daily needs.*

(IDI S-CS01-woman-35)

### 3.4. Changes in Well-Being: Impact of Sed on Stigma, the Effects of Stigma and Quality of Life

**Figure 2:**
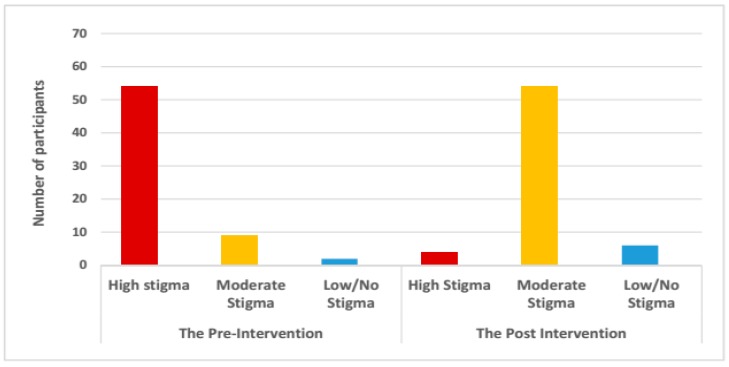
Improvement of stigma level in SED participants based on the profiles (*n* = 65).

SED interventions are expected both to improve participants’ socio-economic status and, through economic transformation, also to improve well-being. In this project we studied changes in well-being through measuring changes in stigma, participation restrictions and quality of life. The profiles data shows that many SED participants previously had a high level of stigma, which was reflected in being scared of leprosy, low self-esteem and limited interaction with community members. The profiles show a reduction of stigma in the way SED participants see themselves and their relationships with others, most moving from a high to moderate level of stigma (see Figure 2). This means they were no longer afraid of leprosy, were more comfortable in telling someone about their disease (though some were still afraid of disclosure), were less ashamed, had more self-esteem and were more willing to interact with neighbours (some still hesitated). A few profiles describe clients with very limited or no stigma. 

The analysis shows a significant reduction in the SSS and PSS total scores of SED participants between the baseline assessment and the final survey (see Table 6). The SSS showed a decrease of 8.5 points between pre- and post-intervention (*p*-value 0.004), the PSS a decrease of 3.6 points (*p*-value 0.0074) and the WHOQOL-BREF an improvement of 4.3 (*p*-value 0.130). 

Comparing the scores of SED participants with the control area and the contact/counselling area shows that their scores differed more (sometimes almost twice as high) than the scores in other areas. Interestingly, the WHOQOL-BREF score appeared to have reduced in the control area (2 points), but this was not statistically significant. 

#### 3.4.1. Impact on Different Types of Stigma and Disclosure Concerns

**Figure 3:**
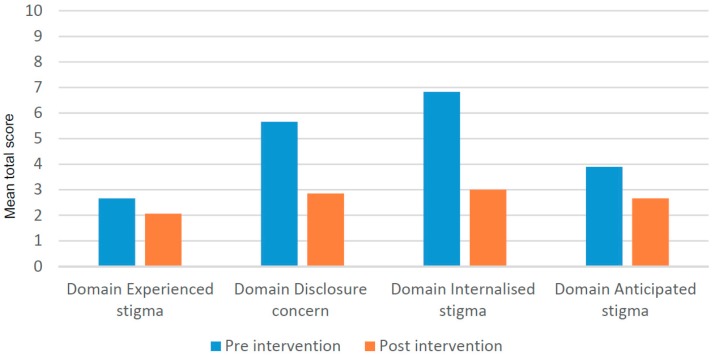
Mean of the four domains scores of the SSS for the SED participants pre-post intervention (*n* = 29).

We now focus on the domains, individual items of the scales that are particularly interesting in relation to the SED intervention and present more qualitative data. Further analysis of the SSS data shows that the SED intervention had an impact on different types of stigma and on disclosure concerns. Figure 3 shows that the largest improvement took place in the internalized stigma and disclosure concerns domains. The experience stigma score reduced slightly (0.59 points or 21%) after the intervention, the disclosure concern score 2.79 points (50%), the internal stigma score 3.83 (56%) and the anticipated stigma score 1.24 points (32%). 

The reduction in domain disclosure and internalized stigma is much higher than the other two domains. Analysis of the qualitative data yielded similar findings, but more importantly it provided insights into how this change was achieved. The trigger that started the cycle of increased self-esteem and therefore increased participation when the person affected by leprosy was offered the chance to join a microcredit scheme. SARI research assistants and KOMIDA staff went to the house of the SED candidate and shared knowledge about leprosy (e.g., that it is curable, the symptoms will disappear and impairment can be prevented) and information about the SARI project and the microcredit scheme (e.g., no collateral and gathering with others). They also motivated and encouraged them to improve themselves through the microcredit. The clients felt accepted by the research assistant and that there were people who still paid attention to them. The provision of knowledge about leprosy, information about the microcredit programme and motivational advice changed the way they saw themselves. The increased self-awareness that they can improve their own life was powerful. By joining economic activities or becoming self-employed they gained the appreciation and respect of the family members and neighbours. It increased their own self-esteem, which then in turn influenced other aspects like participation. Gaining more self-esteem, confidence, and less shame encouraged some of the SED participants to disclose their leprosy status or to worry less about a possible disclosure. Of course, in some cases it is not possible to conceal it due to impairments. Some quotes to illustrate these changes:

My husband has changed. He used to be quiet, didn’t interact or have friends but after SARI came, he has changed. He interacts and doesn’t hide himself any more.

(FGD family counselling-SED client)

Interviewer: *What do you feel after you joined [the SED activity]?*

Interviewee: *Maybe I’ve become better, more courageous. Basically I’ve been better than before.*

Interviewer: *Do you still have any bad feelings about leprosy?*

Interviewee: *I used to, but now I don’t think about it too much.*

Interviewer: *Did you ever talk to anyone else [about your disease]?*

Interviewee: *Some people, yes.*

Interviewer: *What do you talk about?*

Interviewee: *About the disease—the symptoms, the medicines, how to treat it, etc. we basically explained to everyone about what leprosy is*?

(IDI S-CS09-female-43)

#### 3.4.2. Impact on Participation Restriction 

**Figure 4:**
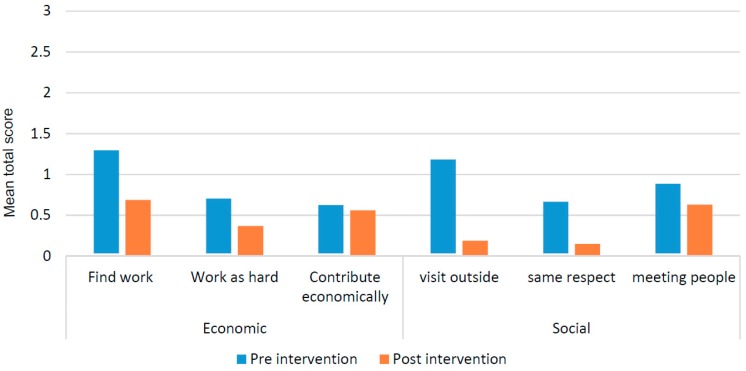
Mean score selected individual items of the PSS by pre- and post-intervention (*n* = 29).

Further analysis of the PSS data shows (Figure 4) an improvement in the opportunities to find work and in working equally hard (same hours, same type of work) as others, however the amount of restrictions to contribute equally to the household remained the same.Also improvements were found on some other items e.g less restrictions to make visits outside the village, receiving the same respect in the community and less restrictions to meeting people in the community. 

In the IDIs and FGDs, many SED participants mentioned that self-confidence helped them to increase their participation in life, which in turn boosted their confidence. In order to run their business SED participants joined training, met other microcredit beneficiaries, and needed to buy things at the market or shop—all of which helped them to interact with their neighbours:

When I had leprosy, I felt numbness so I never went out of the house. After SARI came to my house, I became confident and capable to socialize with other people.

(FGD 2 client SED)

#### 3.4.3. Impact on Quality of Life 

**Figure 5:**
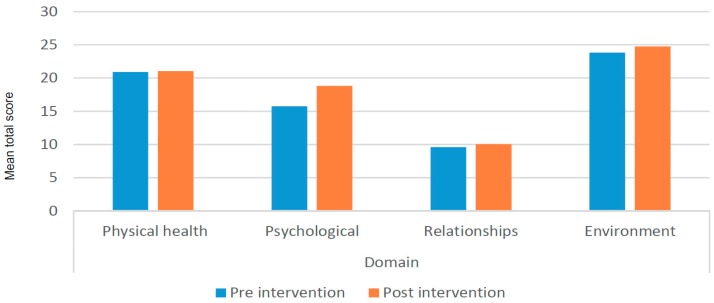
Mean domain scores WHOQOL-BREF by pre-post intervention (*n* = 29).

**Figure 6:**
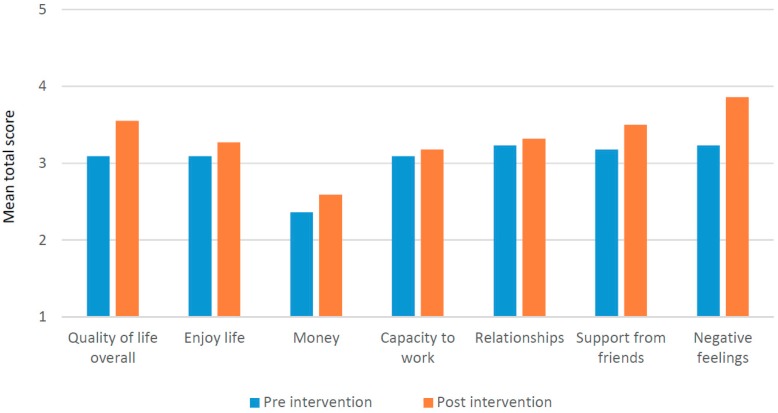
Mean scores selected items of WHOQOL-BREF by pre-post intervention (*n* = 29).

Additional analysis of the WHOQOL-BREF domains shows that most change occurred in psychological health (see Figure 5). Figure 6 shows details of some of the changes in individual items (less negative feelings, more money, better quality of life in general). The quantitative part of the quality of life is addressed in the following section on social transformation. 

### 3.5. Social Transformation: Re-Integration of People Affected by Leprosy into the Community

Following Pati and Lyngdoh [24] the SARI project started from the hypothesis that a microfinance intervention may lead to the participants’ economic transformation, which affects their well-being and could result in overall social transformation. Indeed, we saw that the SED intervention had a positive impact on the socio-economic status of people affected by leprosy and their family and led to a reduction in stigma, fewer restrictions on participation and a better quality of life. Now we are interested in knowing whether a social transformation occurred, and if so what set this in motion. We looked at indicators identified by Pati and Lyngdoh [24] described earlier in the IDIs and FGDs: satisfaction of life, decision-making, health, travel and mobility, recognition and acceptance and capacity (see Table 7). Some participants mentioned they are now happier and more satisfied with their life. Being able to meet their daily needs and knowing more about their disease were important reasons. Several participants—also women and girls, as shown in the quotes—said they are involved in managing the household expenditure and this was easier and more common when women contributed to the income. Many people affected by leprosy remained concerned about their status (e.g., afraid of a relapse or of not being cured) even after being declared cured by a health professional and released from treatment. Data shows that SED participants are more aware of the importance of adhering to medical treatment, and felt more confident about visiting the health centre if they had questions about their disease. The SED participants’ travel and mobility improved as they were less restricted than before, and were motivated to interact with community members and to be involved in community activities. Data also shows an increased recognition and acceptance by household and family members and the wider community. People affected by leprosy worried less about attitudes of neighbours or potential clients. People seemed to be nicer to SED participants and showed more respect, suggesting less anticipated and experienced stigma. The greater capacity of people affected by leprosy led to much improved self-confidence. Overall, the IDIs and FGDs show indicators of social transformation. 

The evidence portrayed in Table 7 shows that economic transformation, improved well-being and social transformation are intricately interwoven and mutually connected. Table 7 confirms socio-economic transformation by showing, for instance, that increased income can lead to satisfaction with life (well-being), which in itself amounts to social transformation. But we also see that more confidence, for instance through greater knowledge, can lead to a larger role in financial decision-making (social transformation), which in turn can lead to improved economic status. This suggests that rather than a linear progression from a microfinance intervention to social transformation, the SED intervention for people affected by leprosy may intervene at any point in the cyclical process connecting economic transformation, increased well-being and social transformation. Increased confidence and self-esteem seem to be major underlying factors contributing to socio-economic transformation of people affected by leprosy.

## 4. Discussion

Microcredit projects are becoming ever more common in Indonesia. Various organizations such as the government (e.g., Bank Negara Indonesia, Bank Rakyat Indonesia, Bank Mandiri), private formal financial institutions (e.g., KOMIDA), faith-based organizations (e.g., Zakat centre, Rumah Zakat) and non-governmental organizations (NGOs) provide microcredit for small-scale businesses [53,54,55]. Marginalized people such as those affected by leprosy or with a disability are often *not* seen as potential clients by formal and informal credit systems [55]. There are a few examples of a specific focus on the most vulnerable social groups (e.g., poorest of the poor, persons with disabilities), but these are exceptional and the programmes are facing serious challenges. In general, many vulnerable groups experience constraints on obtaining credit [53]. This study was set up to realize a ‘proof of principle’ that people affected by leprosy-related stigma could under certain conditions be reliable clients of a microcredit programme. 

Rather than one uniform intervention, the SARI SED intervention comprises a portfolio of activities. Two microcredit schemes and other socio-economic activities were implemented. Both schemes tried to deal with the barriers (e.g., lack of knowledge about leprosy) [19] and opened up opportunities to improve socio-economic status and facilitate a social transformation of people affected by leprosy. The different activities respond to their various needs and are aligned with the locally available resources and programmes, enhancing their sustainability. A strong point is that it that the SED intervention is still running three years after the project ended. Currently, KOMIDA is still recruiting for micro-credits—though to our knowledge not very actively—people with disability and people affected by leprosy. Similarly, the DPO also managed to maintain their micro-credit and related activities. The skills of the DPO personnel have improved substantially over time. Good networking of the DPO with local stakeholders continued and has resulted in various activities for people affected leprosy and people with disability, Districts Social Welfare Office still provide support for grant and material such as tolls and life stock. At the higher level, MOH have commitment to reduce stigma towards people affected by leprosy through national campaign, such as national leprosy day campaign. This condition is accordance to Lockwood et al. general sustainability index of intervention: continues services provision, growing districts local government and National commitment [56]. 

The main purpose of the SED intervention was to improve incomes and reduce stigma and its impacts, such as constrained participation and a lower quality of life. This study confirmed the findings of Ebenso et al. [11], Velema et al. [20] and Wagner et al. [57] of an effect of SED on socio-economic status. Leprosy-related stigma has been studied in depth, but how to reduce it has received less attention [35,58,59]. Velema et al. [20] undertook a literature review in 2008 and found seven programmes that provided socio-economic support to people affected by leprosy [20]. These SED interventions aim to improve socio-economic status mainly through promoting self-employment (e.g., tailoring, cycle repairs). Velema et al.’s review suggests that a well-structured socio-economic intervention might enable persons affected by leprosy to become self-employed, be better off and even self-supporting. Similarly, the median income of SED participants rose more than in the control area, proving that the intervention could improve their socio-economic status. 

The intervention described in this study indicates that SED can help to reduce stigma and participation restrictions and improve the quality of life. Stigma was reduced both in the intervention and the control areas, but mean differences were higher in the former. Restrictions on participation restriction declined in both areas, but was significant in the intervention area but not in the control area. The quality of life of people affected in intervention area was significantly improved, which is a promising result since it declined in the control area. We believe there are two explanations for the observed positive changes in the control areas. First is that the interventions implemented in the sub-districts were intensive and prolonged (over a 2-year period), so a spill-over effect into the sub-districts were interspersed between the interventions areas is quite possible. The second is that the control area subjects were also visited and interviewed twice (baseline and end line) and this in itself may have influenced their outcomes in a positive direction.

Qualitative data also shows that there is often a change in the level of stigma, in line with other similar studies: Shumin et al. found clear evidence that loans have positive social and psychological effect on people living with HIV and AIDS [60]. In India, Gheeta et al. found that an improved socio-economic status improved the quality of life [61]. Chien found a relationship between self-esteem and quality of life among people with schizophrenia [62]. A study by Rao et al. among people affected in India illustrated that increased expectation is bound to lead to changes of people’s perception of their quality of life [61]. Furthermore, the result indicates that people affected by leprosy become more satisfied with their life, have more aspirations for the future, are more aware of their health, access health services, are more involved in decision-making, and experience more recognition and acceptance from people around them. We conclude that these are indicators for a social transformation as defined by Pati [24].

The long-term impact of the SED intervention is the network between the different actors. These new collaborations had a positive impact on the access to care and health sector in general. Village leaders and other key person in the community learnt about leprosy (the symptoms and treatment options) through the SED and were now able to refer new potential cases to the health clinic. But there are many more examples that illustrate the enhanced relationships: a village leader who accompanies a potential new case to clinic, a health worker who asks a village leader to drop some medication to a leprosy patient and who without hesitation agrees to do this, a village leader who encouraged a health worker to come more often to the village to offer support to the patients. These gestures and actions might seem small, but can mean a lot to individuals affected by a stigmatized condition. Future studies could study the effects on the access to care more systematically. 

What is needed to convince organisations that already provide microcredit for small-scale businesses to include people with leprosy or with other stigmatized conditions in their programmes? Based on the experiences of this study we argue that the following three aspects are key. First, time to build relationships. The process of developing and implementing the SED intervention, incorporating views, expectations of the persons affected by leprosy and establishing meaningful collaboration with the DHO, DSWO and other organizations took time. This was necessary to create a sustainable intervention that was embedded in the local context. Second, commitment, all organizations were committed to the cause, inclusion of disempowered and marginalized individuals was part of their vision or mission. Third, knowledge about the condition and about the causes and consequences of stigma among the staff and others involved was key. 

The main limitation of this study is that it has some characteristics of a pilot, although it was not set up that way. The number of SED participants within our cohort (only 29 whereas 66 persons affected by leprosy accessed microcredit) was, for instance, lower than expected and needed to draw firm conclusions. SED was offered to all persons affected by leprosy in the study area (also newly diagnosed), but we realized too late that many (37) were not part of the baseline study. For the quantitative evaluation it was not possible to completely disentangle the effects of SED alone from that of SED combined with counselling. Among the SED participants there were less males compared to the controls and the ‘Counselling Contact’ area and we do not know if this would have any effect on the outcome. We believe that our findings are, nevertheless, important to share with the health community as future SED programs can and should build upon the findings described here to contribute to socio-economic transformation of people affected by leprosy and other marginalized and/or stigmatized groups. 

## 5. Conclusions

In this study we report on the development, implementation and assessment of an SED intervention. Although the number of participants was lower than anticipated, the assessment shows many positive results in terms of socio-economic status, stigma, participation and quality of life. The results are promising and add to the understanding of SED interventions. This study also demonstrates that it has great potential in terms of sustainability and improving the leprosy program. The pledge to ‘leave no one behind’ was a key feature in the discussions leading to the adoption of the Sustainable Development Goals (SDGs) [63]. This study shows that with moderate support a lot is possible for those who were previously left behind. A socio-economic intervention, if embedded and integrated, could provide support to those who are stigmatized by others and themselves. 

## Figures and Tables

**Table 1:** Overview of methods used for data collection.

Approach	Research Methods	Details	Phase	#	Study Population/Object	Stages of the Model of Pati and Lyngdoh
Quantitative	SARI Stigma Scale (SSS)	- Based on the Berger Scale [36] - Adequate cultural validity [37] - 4 Domains: Experienced stigma, Internalized stigma, Disclosure concerns and Anticipated stigma - The SSS asks if a certain experience/feeling occurs (e.g., feel unclean, feel guilty) and if so how frequent (scoring 0–3).	Baseline & Final survey	143	SED clients and people affected by leprosy who lived in the control and counselling/contact area	Well-being
	Participation Scale Short (PSS)	- Shorter version of the Participation Scale which measures restriction in social participation [38,39]. - Participation Scale is validated in several countries [40,41,42] and used in Indonesia before [6]. - The PSS asks whether the interviewee takes part in, for example, family discussions, as their peers do. - The scale has 13 items and a scoring (0–3).				Well-being
	WHO Quality of Life BREF (WHOQOL-BREF)	- 4 Domains; Physical health, Psychological health, Relationships and Environment. - Has been validated, and is widely used in many countries including Indonesia [43,44,45].				Well-being
Qualitative	In-depth interview	- The interviews explored the changes clients experienced in their (i) financial situation, (ii) social situation, (iii) household situation, (iv) health and (v) general well-being. - Most interviews were conducted at the SED client’s house.	Final survey	20	SED clients	Economic transformation, well-being, social transformation
	Focus Group Discussion	- Generally the following topics were discussed: (i) financial situation, (ii) social situation, (iii) household situation, (iv) health, (v) general well-being and (vi) the intervention itself.	Final survey	43	SED clients (2x), family of SED clients, leprosy workers, SED providers (2x), research assistants	Economic transformation, well-being, social transformation
	Profile participant SED	- Written by research assistants, who compared the socio-economic status (of the household and of the business) and stigma before and after the SED intervention. Special attention was paid to being afraid of leprosy, feelings of shame, ideas on concealment/disclosure, willingness to interact with community members.	Final survey	74	SED clients	Economic transformation, well-being, social transformation
	Logbooks, informal interviews, observation	- To assess sustainability of the intervention. - Focus on the demand for socio-economic intervention, the extent to which it addresses the needs of clients, the intensity of the relationship between client and credit provider, embedding in the local system, and the continuation of the intervention one year and two years after the project ended.	Continuously	NA	Study object: sustainability of intervention	N/A

**Table 2:** Overview socio-economic related activities of the SARI project.

Organization	Activity	Intensity of Relation Between Loan Provider and Client	Degree of Regulation	Degree of Disability-Specific (As Opposed to Mainstream)
KOMIDA ^1^	Micro-credit	Low	High	Low
FKDC ^2^	Micro-credit	High	Middle	High/middle
FKDC	Individual loans	Middle	Low	High
DSWO ^3^	Livestock	Low	Middle	High
SARI ^4^ and DSWO	Skills training	Middle	Low	High

^1^ Koperasi Mitra Dhuafa. ^2^ Forum Komunikasi Difable Cirebon. ^3^ District Social Welfare Office. ^4^ Stigma Assessment and Reduction of Impact.

**Table 3:** Beneficiaries of SED SARI project by type of activities.

	Microcredit			Livestock (Goats)	Skills Training
	KOMIDA	FKDC	Individual	DSWO/Others	SARI and DSWO
Persons affected by leprosy	40	20	11	21	52
Community members	242	4	-	-	5
Persons with disabilities	-	8	-	-	-
Total	282	32	11	21	57

**Table 4:** Socio-demographic characteristics of the study participants.

		Quantitative			Qualitative		
		Cohort (*n* = 237)			Profile Observation	In-Depth Interview	FGD
		SED Participants	Control	Counselling-Contact Area	SED Participants	SED Participants	Mixed
*n*		29 (12%)	57 (24%)	57 (24%)	65	20	43
Sex (male)		13 (45%)	37 (65%)	38 (67%)	29 (44%)	5 (25%)	29 (67%)
Age (mean/range) in years		33	38	33	43 (17–70)	20–70	20–60
Marital status (married)		12 (41%)	23 (40%)	21 (37%)	Mostly married	15 (75%)	Mostly married
Education	No schooling	1 (3%)	5 (9%)	1 (2%)	4 (6%)	2 (10%)	-
	>Junior high school	28 (97%)	52 (91%)	56 (98%)	61 (94%)	18 (90%)	30 (70%)
	>College/University	-	-	-	-	-	13 (30%)

**Table 5:** Median family income pre- and post-intervention (IDR in thousands).

	Pre			Post			Difference			
	N	Median	IRQ	N	Median	IRQ	N	Mean	95% CI	
SED participants	26	750	500	29	1000	750	26	554	99	1009
Contact/counselling area	47	600	700	57	1125	1000	47	175	−539	888
Control	50	650	500	57	1000	600	50	363	145	580

**Table 6:** Total score of SSS, PSS and WHOQOL-BREF pre- and post-intervention by area.

		*n*	Pre			Post			Difference			
			Mean	CI 95%		Mean	CI 95%		Mean	CI 95%		*p*-Value ^1^
SSS	SED participants	29	19.03	14.05	24.02	10.59	5.95	15.23	−8.45	−13.94	−2.96	0.0038
	Contact-counselling	57	17.30	13.90	20.69	10.75	8.19	13.31	−6.54	−9.60	−3.48	0.0001
	Control	57	15.42	12.47	18.37	9.79	6.88	12.70	−5.63	−8.92	−2.34	0.0011
PSS	SED participants	29	8.44	4.34	12.55	4.89	1.46	8.32	−3.56	−6.07	−1.04	0.0074
	Contact-counselling	57	6.41	4.51	8.31	3.59	1.76	5.42	−2.82	−4.85	−0.79	0.0074
	Control	57	5.42	3.80	7.04	4.05	2.19	5.92	−1.36	−3.01	−0.29	0.1044
WHO QOL- BREF	SED participants	28	82.59	78.20	86.99	86.91	82.04	91.78	4.32	−1.38	10.09	0.1302
	Contact-counselling	50	84.16	81.17	87.14	88.34	85.05	91.62	4.18	−0.28	8.07	0.0358
	Control	55	85.83	83.36	88.30	83.83	81.32	86.35	−2.00	−5.49	1.56	0.2644

^1^ Paired *t*-test.

**Table 7:** Indicators of social transformation derived from IDIs and FGDs with SED participants.

Indicators	Quote	Examples of What Set Things in Motion:
Satisfaction with life	‘*Well, I am happy now, yeah. My husband and I earn enough [money]*’. (IDI S-CS01-woman-35).	-Being able to meet daily needs -Knowing and sharing correct medical information about leprosy
	*‘I am very happy because I can talk to many people and tell them about the correct understanding of leprosy—that it is curable and cannot infect(other) easily.’* (IDI S-CS14-woman-33)	
Decision-making	*‘My husband once wanted to buy something. I told him not to buy it now, because we need to buy a window for our house. When he wanted to buy a sofa because sometimes we have people coming by our house, I said to him to buy it in credit.’* (IDI S-CS11-woman-33)	-Women have more authority if they contributed to the family income
Health	*‘I think it is going better now. I didn’t take medication very diligently, but now I always take it. It’s like I have motivation.’* (In-depth-S-CS16-man-37)	- Increased confidence of SED clients helped them to visit the healthcare provider for advice
	*‘I was afraid of getting infected again. But [now after visiting the doctor] I understand that once we had medication leprosy will be over. It cannot infect me again. So I am calm after I understand.’* (IDI S-CS06-woman-35)	
	*‘They [health providers] told me not to worry, that the disease would be cured. They counselled me by telling me that I was not the only one suffering from the disease. There were many other people who sought medication for the same disease.*’ (IDI S-CS05-woman-26)	
Travel and mobility	*‘Well, I used to stay at home. Then I thought that it would be better if I joined any kind of social gathering rather than staying at home.’* (IDI S-CS05-woman-26)	-Realization that clients can take action
	*‘Yes, I was invited to become the administrator for national election booth but I gave it to my husband and I became election witness.’* (IDI S-CS06-woman-35)	
Recognition and acceptance	*‘I used to get scared when having the disease. I thought nobody would be willing to buy my merchandise. It turns out that I was wrong, they still want to buy it.’* (IDI S-CT06-man-60)	-Respect because of business -Awareness of wrong assumptions
	*‘We have two groups [for micro-credit gathering]. We were asked to move one group to another house. But they refused: “No, I don’t want. It is more enjoyable to be in this house. I don’t want to move” [house belongs to people affected by leprosy].’* (FGD 2 client SED)	
	*‘Yes, all my neighbours became nicer to me and respect me. ... If there is something like cake, they share it with me. They are nicer and more friendly.’* (FGD 2 client SED)	
Capacity	*‘I had no ability to start anything in the past, but now I can start my small business.’* (IDI S-CT02-woman-21)	-Increased capacity (e.g., because of training) to run a business
	*‘I used to be very down. I was very poor, I hardly had anything to eat. Now, I have built [renovated] a house in the last 2 years. My children’s school fees are paid. My economy is getting better.’* (FGD 1 Client SED)	
